# Genetics of multifactorial disorders: proceedings of the 6th Pan Arab Human Genetics Conference

**DOI:** 10.1186/s12967-016-0854-4

**Published:** 2016-04-20

**Authors:** Pratibha Nair, Sami Bizzari, Nirmal Rajah, Nada Assaf, Mahmoud Taleb Al-Ali, Abdul Rezzak Hamzeh

**Affiliations:** Centre for Arab Genomic Studies, P.O. Box 22252, Dubai, UAE

**Keywords:** Multifactorial disorders, Diabetes, Congenital anomalies, Neurodevelopmental disorders, Cancer, Genetic counseling

## Abstract

The 6th Pan Arab Human Genetics Conference (PAHGC), “Genetics of Multifactorial Disorders” was organized by the Center for Arab Genomic Studies (http://www.cags.org.ae) in Dubai, United Arab Emirates from 21 to 23 January, 2016. The PAHGCs are held biennially to provide a common platform to bring together regional and international geneticists to share their knowledge and to discuss common issues. Over 800 delegates attended the first 2 days of the conference and these came from various medical and scientific backgrounds. They consisted of geneticists, molecular biologists, medical practitioners, postdoctoral researchers, technical staff (e.g., nurses and lab technicians) and medical students from 35 countries around the world. On the 3rd day, a one-day workshop on “Genetic Counseling” was delivered to 26 participants. The conference focused on four major topics, namely, diabetes, genetics of neurodevelopmental disorders, congenital anomalies and cancer genetics. Personalized medicine was a recurrent theme in most of the research presented at the conference, as was the application of novel molecular findings in clinical settings. This report discusses a summary of the presentations from the meeting.

## Background

The 6th Pan Arab Human Genetics Conference, “Genetics of Multifactorial Disorders” was hosted by Sheikh Hamdan Bin Rashid Al Maktoum Award for Medical Sciences (http://hmaward.org.ae) and organized by the Center for Arab Genomic Studies (http://www.cags.org.ae) in Dubai, United Arab Emirates from 21 to 23 January, 2016. The event consisted of a 2 day conference as well as a 1 day workshop on “Genetic Counseling”. The opening ceremony was headed by H.E. Humaid Al Qatami, Chairman of the Board and Director-General of the Dubai Health Authority, and was attended by members of the Board of Trustees of the Sheikh Hamdan Award for Medical Sciences.

The 2-day conference featured 113 research papers that were the basis for seven keynote lectures, 12 oral presentations and 94 posters. The number of contributors to these research papers reached 426 researchers from 35 countries including USA, UK, Germany, Canada, Australia, United Arab Emirates, and Saudi Arabia. These research papers revolved around four main topics: diabetes and other metabolic disorders, genetics of neurodevelopmental disorders, congenital anomalies and cancer genetics. Using modern advances in high throughput sequencing techniques was at the center of many research papers, especially in the context of providing personalized clinical care to patients.

In addition, an exhibition was held on the sidelines of the conference, where seven commercial companies showcased the latest technologies and services offered by them for human genomics and proteomics that are readily applicable for personalized medicine.

## Session 1

In his keynote lecture, Prof. Mark McCarthy from the Oxford Centre for Diabetes provided an overview of how results from GWAS are increasing our mechanistic understanding of Type 2 diabetes mellitus (T2DM) and helping drug discovery achieve better and faster yields [1]. Of the 100 common variants identified by GWAS to be associated with T2DM, techniques such as functional enrichment and using positional candidates have resulted in the discovery of close to 33 functional candidate genes, which can serve as putative therapeutic targets.

The Centre for Arab Genomic Studies (CAGS) showcased its new line of research using the tools of meta-analysis to increase statistical power of studies conducted on Arab populations. “Type I diabetes (T1D) and HLA class II alleles in Arabs” was the focus of the first series of meta-analyses conducted by the Centre. Dr. Abdul Rezzak Hamzeh explained the main findings of these studies and pinpointed the place of Arab data on the global map of ethnic-specific data in the context of T1D [2, 3].

Dr. Anette Gjesing presented some of the work done at the University of Copenhagen in studying the prevalence of Maturity Onset Diabetes of the Young (MODY) variants in Danish women with a history of gestational diabetes (GDM). This study is similar to an earlier study done by the team on the Czech population [4]. The study found that 11 % of diabetes among women with a history of GDM could be explained by MODY variants. These women were also found to be three times more likely to develop diabetes 10-years later. Diagnosis of MODY relies majorly on genetic testing, which is expensive. Dr. Hinda Daggag delivered a talk about her work at the Imperial College of London Diabetes Centre, which involves using Urinary C-Peptide Creatinine Ratio (UCPCR) to diagnose MODY [5]. Her experience with UCPCR testing in the Emirati population seems to suggest that cut-off limits for MODY in this population are lower than the UK limits among pediatric cases and higher among adult cases.

The evolutionary origin of obesity has attracted much popular attention in recent years. Dr. Vadim Stepanov presented the results of his work at the Research Institute for Medical Genetics in Tomsk where his group tried to use genome wide association studies to understand whether obesity is a decanalized phenotype. Their results indicate that observed worldwide frequency spectrum in obesity-associated genes may be, at least partially, explained by the hypothesis of canalization/decanalization of genotype-environment relationships under the pressure of natural selection driven by adaptation to new environment during human migrations.

## Session 2

Prof. Alain Verloes’ keynote lecture on the session on ‘Genetics of Neurodevelopmental Disorders’ was a detailed look into primary microcephalies and how recent genetic discoveries have, to a large extent, blurred the classification of this heterogenous group of disorders [6]. At the beginning of the century, the clinical picture of primary microcephalies was straight forward and simple, with six distinct entities. However, the current pathogenetic classification is a clinical puzzle, with continuum between typical and atypical cases, extensive overlap between clinical diagnoses, and wide genetic heterogeneity. Difficult as it may be, identifying the genetic cause of microcephalies is important because there are prognostic implications for the neurodevelopmental outcome. Equally important is the delineation of the pathogenetic mechanism underlying the condition. Dr. Ganeshwaran Mochida described his work on using CRISPR-Cas9 technology to create a cellular model of microcephaly. His team worked on two different mutations in the ASNS gene leading to a microcephalic syndrome in two unrelated Arab families. By creating ASNS deficient cell-lines using CRISPR-Cas9 genome editing, they were able to show reduced proliferation and increased apoptosis, which was alleviated by asparagine supplementation. Dr. Irma Jarvela presented another case study of a consanguineous Finnish family with non-syndromic intellectual disability, in which whole exome sequencing identified a novel missense variant in the C12orf4 gene. Among local blood donors, this variant was found with a carrier frequency of 1:53, indicating a founder effect and a possible enrichment in this population. Dr. Mohammed Ansari described protein-docking experiments conducted by his group and aimed at understanding the polymerization of alpha-synuclein (SNCA) in synucleopathies. This study enabled the identification of key residues involved in the interaction of the oligomerization assembly, and has the potential to guide therapeutic strategies towards major neurodegenerative disorders involving synucleopathies.

## Session 3

Prof. Stylianos Antonarakis, the current President of the Human Genome Organization delivered the keynote lecture in the session on congenital disorders. He provided a detailed history of personalized medicine and his visualization of genomic medicine for the future. He introduced the ‘genome clinic’, developed in the University Hospitals of Geneva to offer genome diagnostics of Mendelian and complex genetic disorders, for the identification of somatic driver variants in cancer, and to educate the population on risk perceptions and acceptance of genomic medicine. The results from the clinic show a 40 % success rate in identifying the pathogenic variant, with most success coming from Mendelian disorders other than those leading to developmental delay and intellectual disability. The role of sequencing in congenital disorders was exemplified by two lectures, both from the King Faisal Specialist Hospital and Research centre in Saudi Arabia. Prof. Al-Owain described the delineation of a novel syndrome of congenital cataract and abnormal striatum in a consanguineous family, which was found after exome sequencing studies to be linked to KCNA4 mutations. Similarly, Dr. Salma Majid used homozygosity mapping and exome sequencing to identify a novel locus (LACC1) with systemic Juvenile Idiopathic Arthritis [7]. Dr. Tawfeg Ben-Omran from Hamad Medical Corporation in Qatar presented the results of his study on the relation between consanguinity and genetic disorders in the country, which suggests a significant role of paternal consanguinity in the increase in prevalence of genetic conditions, especially autosomal recessive disorders.

## Session 4

Sir John Burn highlighted various important aspects of translational research in genomic medicine. The first of these was exemplified by the ‘BRCA Challenge’project, which aims to pool together, genetic variations and respective phenotypes of BRCA1 and BRCA2 [8]. He also presented the results of the Cancer Prevention Project (CAPP), which aims to test the efficacy of Aspirin as a chemopreventive agent for colorectal cancer. Finally, as an update to developments in diagnostics, the ‘handheld laboratory’ Q-POC was introduced. This device uses nanowire technology to detect DNA sequence variation through changes in electrical current upon binding of sample DNA. The use of disposable cassettes facilitates the analysis of DNA on the spot, in less than 20 min, with low cost and low energy consumption. This device was demonstrated to be an efficient replacement to conventional methods in multiple aspects, such as identifying tumor markers, determining genotype-guided doses of medicine such as Warfarin, or determining drug resistance to Tuberculosis. Prof. Shama Prasada spoke in detail on the use of epigenetically modified genes as diagnostic markers in cancer; as an example, he illustrated how different levels of aberrant methylation in multiple genes correlates with different stages of cervical cancer. In addition, he shared his recently published results on the tumor suppressor function of a previously untested gene, Double C2-like Domain β (DOC2B), which encodes a calcium sensor involved in neurotransmitter release and exocytosis. Due to the hypermethylated state and low expression of DOC2B in the pre-malignant stage of cervical cancer, it may potentially be used as a reliable marker for early diagnosis [9, 10]. Dr. Abeer Alsaegh addressed various challenges faced in the implementation of a cancer genetics service in Oman, offered by Sultan Qaboos University Hospital. She spoke on the recent efforts to organize the details of patients referred for cancer services through the construction of a database, and shared statistical data of mutations in specific genes for breast and colon cancers. She highlighted the high consanguinity rate in Oman, and the extremely high rate in those who are referred for familial cancer testing. She described the significant reluctance of Omani individuals and families in participating in genetic counselling and testing, which should be addressed through raising awareness and promoting education in the general public.

## The genetic counselling workshop

On the third day, a specialist workshop was delivered by Dr. Christine Patch from Guy’s Hospital, London and Dr. Anna Middleton, from Wellcome Trust Sanger Institute. The workshop introduced the basic principles of genetic counseling and explored different ethical issues in counselling using role plays and real-life case studies. Some of these case studies discussed issues such as genetic counseling for developmental disorders, disability, prenatal genetic diagnosis, and pre-symptomatic testing.

## Conclusions

Pan Arab Human Genetics Conferences (PAHGC) are held biennially to provide a common platform to bring together regional and international geneticists to share their knowledge and to discuss common issues, and the sixth version of this series was no exception. As well as offering a great opportunity for young and more experienced scientists to mingle, the 6th PAHGC contributed significantly to the advancement of human genetics and its applications in general. As a matter of fact, the event focused on the most pressing issue of our time, personalized medicine. Obviously, the hottest topic in human genetics is the great speed at which molecular analyses can be performed these days, and researchers all over the world are trying to “translate” these advancements into optimal treatment for patients. This issue featured repeatedly throughout the conference and many brilliant ideas were exchanged in this regard. The organizers also wished to shed light on the challenges faced by the medical sector upon trying to deliver this wealth of information to the general public, and hence the chosen topic of the workshop was “genetic counselling”. In fact, there is a dire need for more qualified genetic counsellors in this part of the world and efforts such as the conference and its workshop are mere steps towards addressing this problem.
